# A case of postoperative spontaneous intussusception after laparoscopic low anterior resection for rectal cancer

**DOI:** 10.1186/s40779-016-0087-0

**Published:** 2016-06-18

**Authors:** Young Wan Kim

**Affiliations:** Department of Surgery, Division of Gastrointestinal Surgery, Yonsei University Wonju College of Medicine, Wonju, Korea

**Keywords:** Rectal neoplasms, Intussusception, Postoperative complications

## Abstract

**Background:**

Intussusception refers to a condition in which a segment of the intestine invaginates into the lumen of an adjacent segment of the intestine. Postoperative intussusception after gastrointestinal surgery is an uncommon clinical condition, and there is only one case report of small bowel intussusception after rectal cancer surgery. Here, we report a case of spontaneous small bowel intussusception following laparoscopic total mesorectal excision for rectal cancer.

**Case presentation:**

A 56-year-old female military officer was referred to the Colorectal Surgical Department for mid-rectal cancer, 8 cm from the anal verge. The patient underwent laparoscopic low anterior resection and diverting loop ileostomy. On postoperative day 3, the patient complained of vomiting and abdominal pain, and a follow-up abdomino-pelvic computed tomography scan showed an ileo-ileal type intussusception. After two days of surgical observation, her clinical symptoms were not resolved. The patient underwent exploratory laparotomy. On exploration, intussusception was found 40 cm proximal to the loop ileostomy site. Segmental resection of the ileum was carried out, and there was no pathological leading point on the resected ileum. The patient was discharged on postoperative day 14 after the second operation and has remained in good health for two years.

**Conclusion:**

We present a case of spontaneous small bowel intussusception after laparoscopic total mesorectal excision for rectal cancer that was treated by surgical resection 5 days after the index surgery.

## Background

Intussusception refers to a condition in which a segment of the intestine invaginates into the lumen of an adjacent segment of the intestine. Intussusception in pediatrics ranks second to appendicitis as a cause of acute abdominal surgery, and most cases are idiopathic. However, the incidence of adult intussusception is approximately 5 % of all cases of intussusception, and most cases have an underlying cause, such as malignancy [1]. Postoperative intussusception after gastrointestinal surgery is an uncommon clinical condition with a reported incidence of less than 0.1 % in patients undergoing gastric resection [2], and Atolagbe et al. [3] recently reported a rare case of retrograde intussusception of the roux limb following laparoscopic Roux-en-Y gastric bypass surgery. There is only one case report of small bowel intussusception after rectal cancer surgery [4]. Here, we report a case of spontaneous small bowel intussusception after laparoscopic total mesorectal excision for rectal cancer.

## Case presentation

**Fig. 1:**
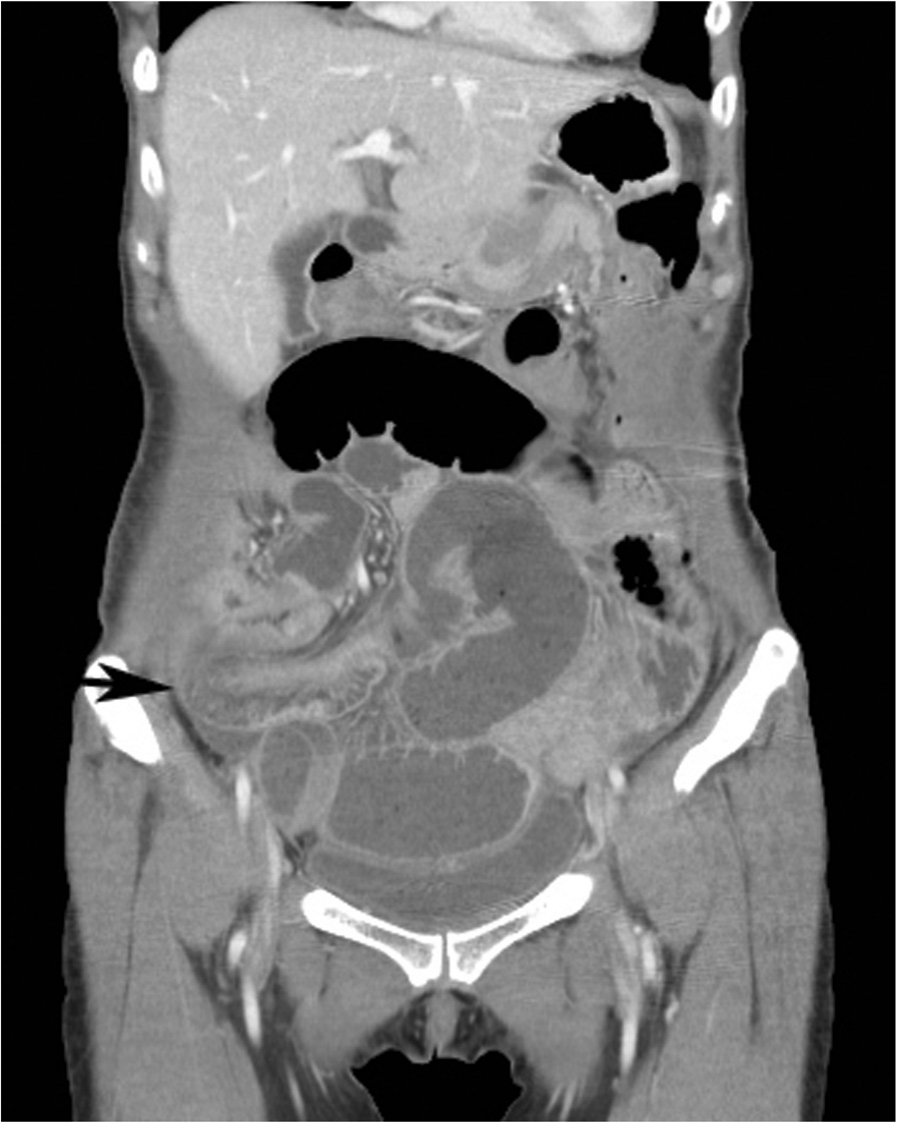
A follow-up abdominal computed tomography (CT) scan after laparoscopic low anterior resection for rectal cancer on postoperative day 3. A coronal image showing ileo-ileal type intussusception (*black arrow*)

**Fig. 2:**
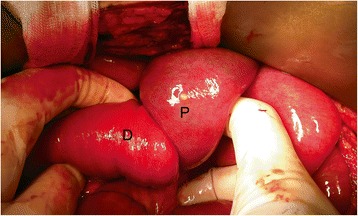
Exploratory laparotomy for intussusception. The proximal ileum (P, intussusceptum) invaginated into the distal segment (D, intussuscipiens)

**Fig. 3:**
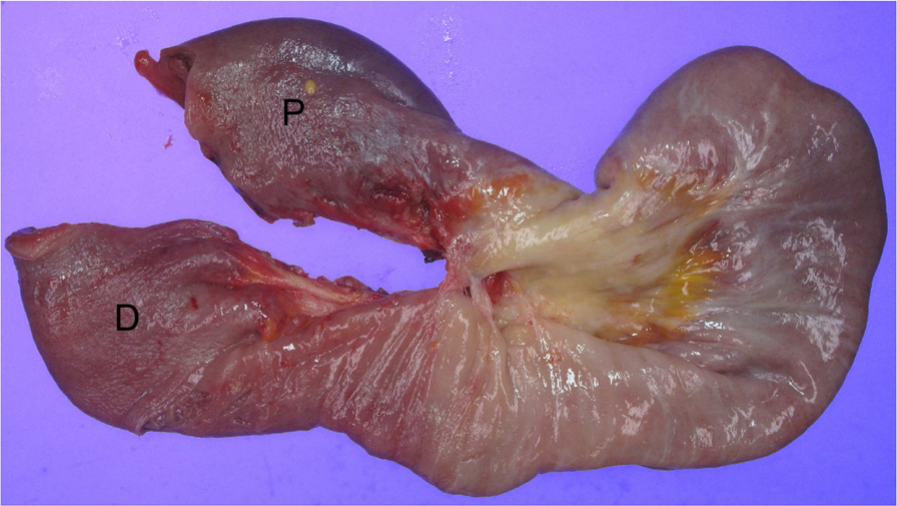
Specimen examination after laparotomy. There was no pathological leading point on the resected ileum

A 56-year-old female military officer was referred to the Colorectal Surgical Department for mid-rectal cancer 8 cm from the anal verge. The patient had no past medical history. Her physical examination and routine laboratory studies were unremarkable. Her carcinoembryonic antigen level was 5.93 ng/ml, and an abdomino-pelvic computed tomography (CT) scan showed no intra-abdominal metastasis. Pelvic magnetic resonance imaging showed a clinical T_2_N_0_ tumor. The patient underwent laparoscopic low anterior resection and diverting loop ileostomy. The operation time was 310 min. The pathologic results showed that the adenocarcinoma had infiltrated the proper muscle layer (T_2_) with no lymph node metastasis (0/17). On postoperative day 3, the patient complained of vomiting and abdominal pain, and a follow-up abdomino-pelvic CT scan showed an ileo-ileal type intussusception (Fig. 1). Two days of surgical observation were unsuccessful, and her nausea and abdominal pain continued. The patient then underwent exploratory laparotomy. On exploration, intussusception was found 40 cm proximal to the loop ileostomy site. The proximal ileum (P, intussusceptum) had invaginated into the distal segment (D, intussuscipiens), and segmental resection of the ileum was carried out (Fig. 2). There was no pathological leading point on the resected ileum (Fig. 3). The patient recovered uneventfully and was discharged on postoperative day 14 after the second operation. The patient has remained in good health for two years.

## Conclusions

Postoperative intussusception is a distinct clinical condition, and in terms of rectal surgery, only one study to date has described small bowel intussusception following laparoscopic low anterior resection, which was in a 75-year-old man [4]. The pathogenesis of postoperative intussusception is unclear, and pathologic examination usually reveals no identifiable cause or pathologic leading point following surgery for the postoperative intussusception [5], as in our case. There are several hypotheses regarding potential causes including extensive handling of the small bowel, prolonged ileus, increased abdominal pressure, peristalsis, and/or fibrous adhesions around the suture site [6]. The clinical manifestations are similar to those of postoperative small bowel obstruction. Nausea, vomiting, and abdominal pain are frequent symptoms, but a palpable mass and/or currant jelly stools are infrequent [5]. It is difficult to differentiate postoperative intussusception from paralytic ileus in patients experiencing mild clinical symptoms such as abdominal discomfort and failure to pass a flatus. Indeed, Hussain, et al. [4] warned of delayed diagnosis of postoperative intussusception. Their case was diagnosed 22 days after the index surgery because the patient presented with non-specific abdominal symptoms such as obstipation, mild abdominal bloating, and emesis. In our case, the diagnosis was made on postoperative day 3, based on CT scans. Intussusception can be diagnosed using imaging studies such as CT or abdominal ultrasonography (US). Given that there is a postoperative abdominal wound, CT may be more useful than US. A US probe may cause severe wound pain, and the surgical wound may disturb the ultrasound [7]. Thus, to prevent a delay in diagnosis, a high index of suspicion is required during the postoperative period. Once suspected, CT scans can be beneficial for differential diagnosis. Differential diagnosis with paralytic ileus is difficult in patients with small bowel obstruction, but vomiting and abdominal pain are rare in patients with paralytic ileus. CT scans can reveal whether a complete small bowel obstruction is present. In addition, adhesion-related obstructions and non-adhesional pathology can be observed on CT scans. CT estimates the presence of strangulation with sensitivity and specificity over 90 % and a negative predictive value of approximately 100 % [8]. The treatment is usually surgical resection. However, spontaneous reduction has been reported in some cases [9]. In our case, two days of surgical observation were not successful, and the patient’s symptoms were not resolved. Given that a natural course of postoperative intussusception is not clearly defined, surgical treatment should be decided upon carefully. The prognosis is usually excellent unless the patient has a pathologic condition such as polyps or lipoma [10]. In our institution, a protective stoma was not routinely constructed. Low-level colorectal anastomosis (<5 cm from the anal verge), the presence of air leakage, and an incomplete donut were the main indications for the de-functioning ileostomy [11].

In summary, we present a case of spontaneous small bowel intussusception after laparoscopic total mesorectal excision for rectal cancer that was treated by surgical resection 5 days after the index surgery.

## Abbreviations

CT, Computed tomography; US, Ultrasonography
